# 3D Exoscopes in Experimental Microanastomosis: A Comparison of Different Systems

**DOI:** 10.3390/life13020584

**Published:** 2023-02-19

**Authors:** Ahmad Hafez, Roel Haeren, Justiina Huhtakangas, Ville Nurminen, Mika Niemelä, Martin Lehecka

**Affiliations:** 1 Department of Neurosurgery, Helsinki University Hospital, P.O. Box 266, Fin-00029 Helsinki, Finland; 2 Department of Neurosurgery, Maastricht University Medical Center, Postbus 5800, 6202 AZ Maastricht, The Netherlands

**Keywords:** Exoscope, comparison, bypass, ORBEYE, VITOM, AEOS, KINEVO

## Abstract

*Background*: In recent years, three-dimensional exoscopes have been increasingly applied in neurosurgery. Multiple exoscopic systems are available, all offering specific features. In this study, we assessed practical and visualization performance of four different exoscopic systems in a highly challenging microsurgical procedure, and evaluated whether these affected the quality of work. *Methods*: We included four different exoscopes: Olympus ORBEYE, Zeiss KINEVO, Storz VITOM, and Aesculap AEOS. With each exoscope, ten experimental bypass procedures were carried out on chicken wing vessels at a depth of 3 cm. We evaluated the quality of the anastomoses, the practical considerations for the setup of the exoscopic systems, and the visualization quality by tracking the number of unnecessary movements. *Results*: All included exoscopes enabled us to perform the bypass procedures with mostly adequate or excellent results. Surgically, the most pronounced difference between the exoscopes was the duration of the procedure, which was mainly due to the number of unnecessary movements. Practically, the exoscopes differ highly which is important when considering which exoscope to apply. *Conclusions*: This is the first study comparing different exoscope systems while performing the same challenging microsurgical procedure. We found major practical differences between the exoscopes that determine the suitability of an exoscope based on the demands and conditions of the surgical procedure. Therefore, preprocedural practical training with the exoscope is required.

## 1. Introduction

In recent years, three-dimensional (3D) exoscope systems have consistently been reported to form a potential alternative to the conventional operating microscope in neurosurgery [1,2,3,4,5,6,7,8]. Exoscopes visualize the surgical field from above with a 3D digital camera, and project this view on the surgical field on a 3D monitor. This ocular-independent visualization of the surgical field enables more comfortable and unconstrained working posture for the surgeon and allows a larger range of movements within the surgical field [1,3,9]. Moreover, the 3D visualization of the surgical field is similar for all participants of the surgery, which potentially improves surgical and anatomical education [3,10,11].

Besides these benefits, previous studies comparing exoscope systems with operating microscopes demonstrated that image quality, illumination, range and quality of magnification and focus were at least comparable [3,11,12,13,14,15,16,17,18,19]. Recent studies also reported on the successful incorporation of numerous fluorescence-based imaging tools such as 5-aminolevulinic acid and indocyanine green video-angiography [2,18,20,21]. Based on these findings, the exoscope has been suggested to become the primary visualization tool in neurosurgery in the future [4,8,9,21,22].

Multiple exoscopic systems are currently available, all offering specific features, and thereby having their own strengths and limitations. To stimulate their evolution, it is relevant to compare these exoscope systems. Apart from one study reviewing features of the various exoscopic systems [4], there are no studies that have evaluated different exoscopes in practice. In this study, we compared the performance of four different exoscopic systems in a highly challenging microsurgical procedure (i.e., an experimental bypass at a depth of 3 cm (cm)). We aimed to assess practical and technical features of the systems, and evaluate whether they affected the quality of work.

## 2. Materials and Methods

### 2.1. Ethical Statements

Approval of this study by the local Institutional Review Board was not required according to national legislation.

### 2.2. Exoscope Systems

For this study, we aimed to evaluate five commercially available exoscope systems that have been reported on most frequently in recent literature. These five exoscopes systems are: Olympus ORBEYE (Olympus, Tokyo, Japan, further referred to as ORBEYE), Zeiss KINEVO (Carl Zeiss Meditec AG, Jena, Germany, further referred to KINEVO), Storz VITOM^®^ 3D (Karl Storz SE and Co. KG, Tuttlingen, Germany, further referred to as VITOM), Aesculap AEOS (Aesculap Inc., Center Valley, Pennsylvania, USA, further referred to as AEOS), and the Synaptive Modus V (Synaptive Medical, Toronto, ON, Canada). However, we had to settle with four systems only as due to COVID-19 pandemic we had to leave the Synaptive Modus V out. For the projection of the procedure with the ORBEYE, KINEVO and AEOS systems, we used the same monitor, i.e., a SONY monitor (55 inch, 3D 4K (resolution: 3840 × 1080). A STORZ monitor (32 inch, 3D 4K, (resolution: 3840 × 2160)) was used for the procedures performed with the VITOM exoscope. At the time we performed the procedures with the VITOM exoscope, the larger SONY monitor screen was not yet available at our center.

### 2.3. Materials

The experimental bypass procedures were carried out using a self-made Styrofoam model of a human head, as previously described [1]. The model contained a 2 cm working field at a depth of 3 cm, with an opening of 3 cm on the surface. We prepared chicken wing vessels with a median diameter of 1 mm (mm) for experimental, end-to-side anastomoses. For these procedures, the following surgical instruments were used: two SuperBypass forceps (SB-1607, Takayama Instruments Inc., Muranaka, Tokyo, Japan), microscissors (Mutoh America Ltd., Natick, MA, USA), a Yasargil Aneurysm Clip System (Aesculap, Inc., Center Valley, PA, USA), 2 temporary clips, and a 12-0 polyester suture needle (Ningbo Medical Needle Co. Ltd., Ningbo, China).

### 2.4. Anastomosis Procedure

We performed end-to-side anastomoses using 20 interrupted sutures evenly distributed along the vessel orifice at a depth of 3 cm, as described previously [1,10]. We performed a total of ten bypass procedures with each exoscope system. All anastomoses were performed by A.H., who has performed over 3000 experimental bypasses including around 400 using an exoscope. We opted for an experienced surgeon to limit the effect of factors that are related to the learning curve and specifically address the objective how an experiences neurovascular surgeon adapts to working with different exoscopes [23].

### 2.5. Outcome Parameters

First of all, we evaluated the quality of the anastomoses using the TSIO scale (see Table 1) [10]. This scale encompasses assessment of the duration of the procedure (T), distribution of the stitches (S), whether the thread is hidden within the vascular wall (I), and the orifice width (O). A favorable TSIO score is defined as 3 or 4 points on the TSIO scale, whereas an unfavorable TSIO score is defined as 0–2 points. Secondly, we analyzed the visualization quality of the exoscopes based on hand movements. The ease of performing the hand movements for this microsurgical procedure depends on a sufficiently large field of view and more importantly the depth of field (i.e., the range between the nearest and the farthest objects that are in acceptably sharp focus) while using high magnification, as we described previously [9]. A reduced field of view or limited depth of field will increase the total number of focus and magnification adjustments which on itself increases the duration of the procedure. More importantly, the number of hand movements, in particular of unnecessary hand movements, would be higher in case the visualization quality is reduced by a limited wideness and/or depth of field. Hand movements were classified as necessary movements or unnecessary movements. Necessary movements were defined as hand movements that accomplished the goal that was intended with the movement (e.g., grabbing the needle, tying a knot). On the contrary, unnecessary movements were defined as hand movements that did not accomplish the goal that was intended by the movement. To understand the intention of the surgeon’s movements and to define them as necessary and unnecessary, the performing surgeon (A.H.) showed and explained his strategy and chronology of hand movements for performing anastomoses. Furthermore, the performing surgeon and the video assessors (J.H. and V.N.) watched, evaluated and discussed multiple videos of his anastomoses performed with a microscope. Besides this specific assessment training, both assessors were previously trained in performing microanastomoses by the performing surgeon. Using video recording of the anastomosis procedure with the different exoscopes, the two assessors thereafter independently counted necessary and unnecessary movements. Lastly, the performing surgeon (A.H.) and/or video assessors (J.H., V.N.) subjectively rated image quality, illumination, and ergonomics.

### 2.6. Statistical Analyses

Statistical analysis was performed using IBM SPSS Version 25 (IBM Corp., Armonk, NY, USA). Data is presented as mean and standard deviations (SD). Pearson χ^2^ test was used to predict the relationship between variables and the different exoscope systems. A *p* value of <0.05 was considered to be statistically significant.

## 3. Results

### 3.1. Practical Setup and Subjective Evaluations

**Figure 1:**
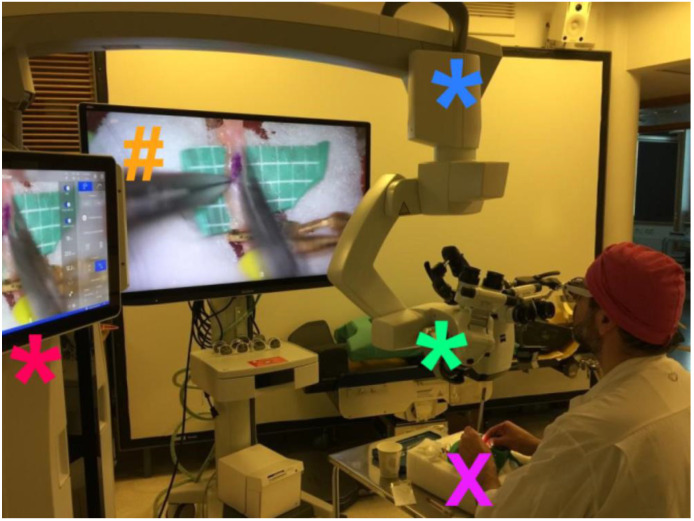
Experimental setup for KINEVO exoscope. This image depicts the practical setup for the experimental anastomoses using the KINEVO. The surgical field (**X**) is visualized by the camera that is included in the operational microscope housing (*). This housing is consequently relatively bulky compared to the other exoscopes’ camera housings. Due to this bulky housing, we had difficulty positioning the exoscope such that it did not obstruct the surgeon’s view on the three-dimensional monitor (**#**). This is further complicated by the arm of the exoscope (*) that comes from above compared to a sideways connected arm in the other exoscope systems. An advantage of this arm position and the length of the arm, is that it does not affect sterility of the surgical field. The surgeon nevertheless experienced a relaxed posture while performing the anastomoses in sitting position. The exoscope operational base (*) is also rather bulky as it is also the base for the microscope systems. The operational base included two monitors, one of them displaying a three-dimensional image of the surgical field. * exoscope operational base; * exoscope camera; * fixation arm; **X** styrofoam model of a human head for the experimental anastomoses; **#** three-dimensional monitor.

**Figure 2:**
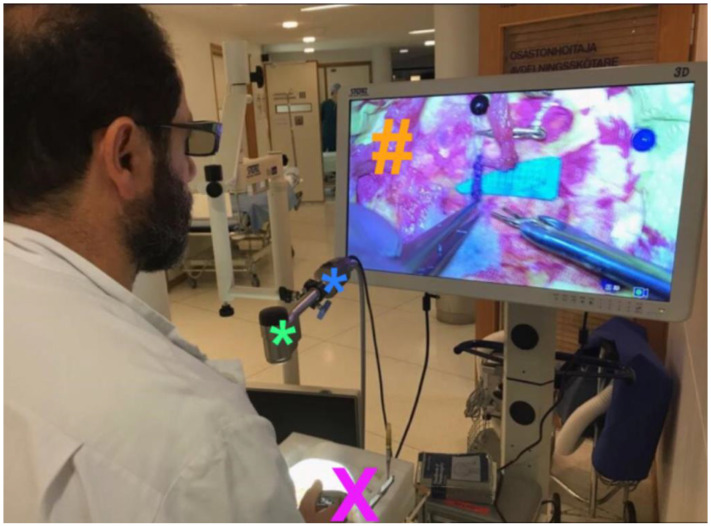
Experimental setup for VITOM exoscope. In this image, the setup for the experimental bypasses using the VITOM exoscope is shown. The surgical field is visualized with the exoscope camera (*) placed directly above the surgical field (**X**). The surgeon himself is performing the anastomoses in a relaxed sitting position while watching its movements on the three-dimensional monitor (**#**) using three-dimensional glasses. The monitor was placed in the sight-line of the surgeon which was not obstructed by the camera, as the camera is rather small. The applied three-dimensional monitor was relatively small and therefore placed more closely to the surgeon when compared to the other exoscope systems. The exoscope camera is attached to a fixation bar with a relatively small length (*) that would complicated sterile procedures. The exoscope operational base (*) is integrated within the housing of the applied monitor. * exoscope camera; * fixation bar; **X** styrofoam model of a human head for the experimental anastomoses; **#** three-dimensional monitor.

**Figure 3:**
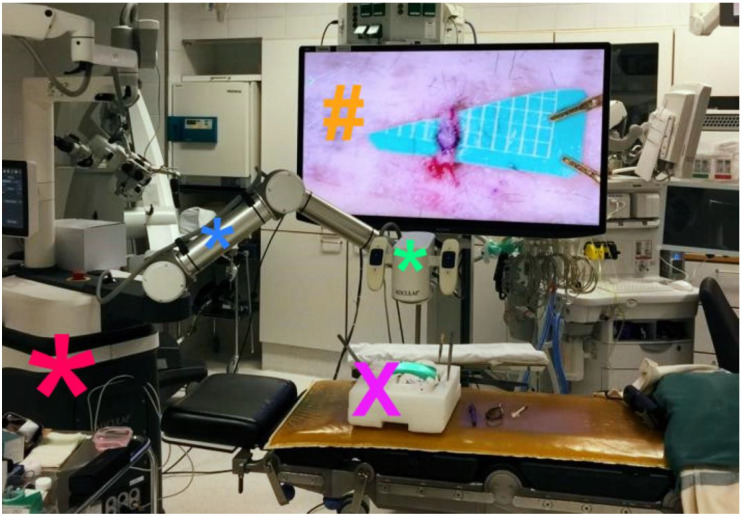
Experimental setup for AEOS exoscope. This picture illustrates the practical setup of the AEOS exoscope to perform experimental anastomoses. The surgical field (**X**) is visualized by the exoscope camera (*). The camera housing also contains a handgrip on each side that enables manual adjustments of magnification, focus and camera position. Despite these handgrips, the camera is relatively small and did not obstruct the surgeon’s view on the three-dimensional monitor (**#**). The robotic exoscope arm (*) is placed sideways and contains three joints to enable horizontal, vertical and spherical movements of the camera. The arm is of adequate length to position the exoscope operational base (*) sufficiently outside the surgical field, in particular in case of sterile procedures. * exoscope operational base; * exoscope camera; * fixation arm; **X** styrofoam model of a human head for the experimental anastomoses; **#** three-dimensional monitor.

**Figure 4:**
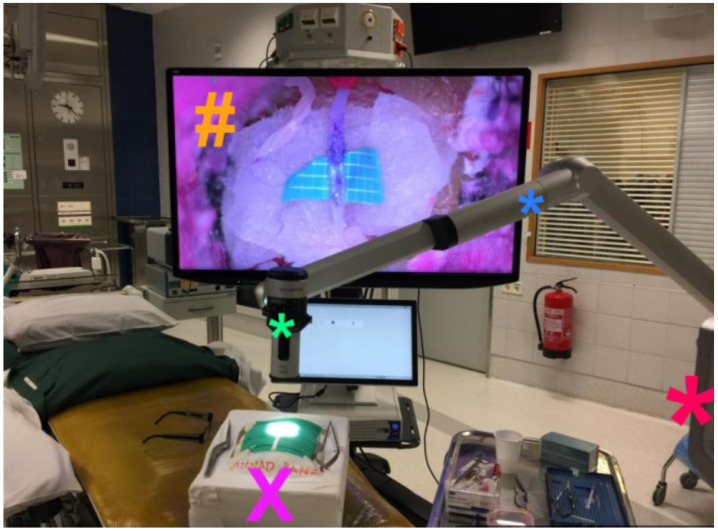
Experimental setup for ORBEYE exoscope. Here, the practical setup for experimental anastomoses using the ORBEYE exoscope system. The surgical field (**X**) is visualized by the exoscope camera (*) and displayed on the three-dimensional monitor (**#**). The surgeon’s sight-line on the monitor is not obstructed by the small camera. The exoscope arm (*) is located on top and directed sideways of the camera, not obstructing the surgeon’s view on the monitor. The length of the arm is adequate to enable sterile procedures while the exoscope’s operational base (*) is placed sufficiently distant of the surgical field. * exoscope operational base; * exoscope camera; * exoscope arm; **X** styrofoam model of a human head for the experimental anastomoses; **#** three-dimensional monitor.

All exoscope systems were placed above the Styrofoam model to perform the anastomoses. Positioning of the exoscopes and monitor was slightly different for each system and more time consuming than expected. Particularly, we noted that the KINEVO was rather bulky as an exoscope systems compared to the other three exoscopes thereby easily obstructing the in-line view on the 3D monitor (Figure 1). As a consequence, it was more difficult to find the ideal positioning of the KINEVO. As mentioned, we applied a smaller 3D monitor for the procedures with the VITOM exoscope. To compensate for this difference, we positioned the STORZ monitor more closely to the surgeon. Preparations for each system were also different. For example, the VITOM exoscope system was attached to a fixation bar (Figure 2), while the other three camera systems were directly coupled to an adjustable and moveable exoscope arm (Figure 1, Figure 3 and Figure 4).

The working distance of the exoscope to the surgical field was different for all exoscopes which was related to the various features regarding focal distance in combination with optimal magnification settings. A foot pedal was used to alter magnification using the KINEVO, ORBEYE (Figure 3) and AEOS (Figure 4), while magnification was adjusted manually using the VITOM. Focus adjustments were applied using the foot pedal for the KINEVO and ORBEYE, whereas auto-focus was used for the AEOS and focus was adapted manually using the VITOM. Foot pedal adjustments as well as the auto-focus function of the AEOS worked well. Although the manual adjustments for the VITOM were easily applied, they require the surgeon to move one hand from the operative field. This interferes with the procedural movements and flow. Hence, these manual adjustments were reduced to a minimum by predefining optimal focal and magnification settings before initiation of the anastomosis procedure.

Our surgeon (A.H.) did not experience any differences regarding ergonomics, with the surgeon in a sitting position for the bypass procedures while having a direct and unobstructed in-line view on the monitor. Subjectively, illumination was of sufficient quality and more or less comparable between most exoscopes. The image quality was evaluated best for the KINEVO which was considered the sharpest image. In contrast, the ORBEYE system was found to be slightly troublesome to distinguish between color contrasts, in particular reddish colors.

### 3.2. Bypass Procedures

A total of ten bypass procedures were performed with each exoscope system. A favorable TSIO score for the bypass procedure was noted in 7 out of 10 procedures when using the AEOS exoscope, 6 out of 10 procedures when using the ORBEYE system, in 3 out of 10 when using the VITOM system, and in none of the 10 procedures in which the KINEVO system was applied (Table 2). None of the bypass procedures were ascribed a maximum TSIO score of 4, as the duration of all procedures was above the 20 min cut-off value. The mean duration (±SD) of the procedures per exoscope system in order of shortest to longest duration were: AEOS (26.2 ± 1.8 min), VITOM (27.7 ± 2.7 min), KINEVO (29.5 ± 1.8 min), and ORBEYE (34 ± 3.1 min). We did not find any trend for a shorter duration following more procedures performed on either exoscope. The mean duration of all procedures was 29.5 min (±4.0 min, range 21–40). A cut-off value of 29 min was therefore used for statistical evaluation of variables related to affect procedure duration (see below).

### 3.3. Unnecessary Movements

Unnecessary movements were noted in all procedures, ranging from 20 to 69 unnecessary movements. Mean number (±SD) of unnecessary movements were 25.6 (±5.4) for the AEOS, 27.0 (±4.6) for the VITOM, 32.5 (±9.5) for the KINEVO and 49.2 (±11.1) for the ORBEYE, which differed significantly (*p* < 0.001, Table 3). The overall mean number of unnecessary movements was 33.57 ± 12.3. Based on this, we used a cut-off value of 34 to distinguish between acceptable and unacceptable number of unnecessary movements. When applying this cut-off value, we found that bypass procedures included an acceptable number of unnecessary movements in 9 out of 10 procedures using the VITOM and AEOS exoscope, and in 8 out of 10 procedures when the KINEVO system was applied. When using the ORBEYE exoscope, an acceptable number of unnecessary movements was noted in 1 out of 10 procedures.

An acceptable number of unnecessary movements was strongly correlated to the duration of the bypass procedures of less than 29 min (*p* = 0.002). Adequate quality of the stitch distance and distribution was also related to (*p* = 0.038) reduced duration of the procedure. Unnecessary movements and all other TSIO variables were otherwise not significantly correlated.

## 4. Discussion

This is the first study comparing different exoscope systems while performing the same highly challenging procedure. For the surgical task, we found that the most pronounced difference between the exoscopes was the duration of the bypass procedure, which on itself was mainly related to an increased number of unnecessary movements. Regarding the technical aspects of each exoscope, we noted that image quality, focus, magnification, and illumination features were adequate for all systems. Each exoscope comes with specific advantages and limitations which is important when considering which exoscope to use or how prepare the practical setup.

### 4.1. Technical Features

In this study, we included experimental bypass procedures to challenge the technical visualization features of the exoscope systems. Firstly, experimental bypass procedures require an imaging system that accommodates rapid magnification and focus adjustments, and offers optimal wideness and depth of field. Subjectively, we found the image quality was more than adequate for all exoscope systems, while we considered image quality was best for the KINEVO system, whereas we had more difficulty with color contrasts using the ORBEYE. To objectify our experiences, we included the TSIO scores for the assessment of anastomosis quality and the number of unnecessary movements. Image quality, focus, and magnification adjustments as well as wideness and depth of field may heavily affect TSIO scores and the number of unnecessary movements [10,19]. The duration of the anastomosis procedure was overall relatively long (mean duration varying between 27.7–34.0 min), regardless of the applied exoscope system. This is in line with our previous experience with exoscope systems, as well as other previous studies comparing exoscope systems with operating microscopes [1,5,6,9,13,14,24]. Importantly, none of the procedures was finished within 20 min, which is the cut-off value within the TSIO scale based on closing the bypass as quickly as possible to limit secondary ischemic brain injury [10]. This relatively poor result regarding the duration of the procedure is most likely due to the surgeon not being fully acquainted with the exoscope system. For example, it seems plausible that it takes time to get used to the different type of visualization (i.e., digital visualization), as well as being efficient in adjusting the exoscope such that visualization is optimal. Previous studies indeed described a reduction in procedure duration in consecutive cases, suggesting that the prolonged duration was related to surgeon’s experience with the exoscope [1,24]. Moreover, previous studies suggested that the number of focus and magnification adjustments is increased when using exoscope systems compared to operating microscopes [5,9,13,14]. In this study, we did not prospectively collect the number of focus and magnification adjustments during the procedures. We also did not perform a post-hoc evaluation of the number of these adjustments using the video recordings of the procedures. On the one hand, comparing these numbers seems unfair as we applied different strategies for focus adjustments (foot-pedal, manually and auto-focus), and adjustments may easily go unnoticed as differences are very small. Importantly, we consider it unlikely that the overall prolonged duration of the bypass procedures was related to the surgeon’s skill as he performed over 3000 experimental bypass procedures. This surgeon usually finishes around 70–80% of his experimental bypass procedures with interrupted sutures within 20 min when using the operating microscope [1]).

We found that favorable TSIO scores were achieved most frequently with the AEOS (7/10) and ORBEYE (6/10) exoscopes, whereas VITOM (3/10) and KINEVO (0/10) performed less. The AEOS and VITOM exoscopes also outperformed the other systems with regards to the time needed to perform the anastomosis. For the AEOS exoscope, this may be related to the auto-focus function that enabled rapid focus adjustment. The number of adjustments with the VITOM exoscope were reduced to a minimum as they had to be performed manually which may have reduced the overall procedures’ durations. Interestingly, the number of unnecessary movements was not affected by the limited number of focus and magnification adjustments, suggesting that the settings were optimized before procedures were performed, with adequate image quality, width and depth of field.

On the contrary, none of the procedures performed using the ORBEYE were below the average time of all exoscope systems. This was probably due to the high number of unnecessary movements, which was highest with the ORBEYE and almost twice the number of unnecessary movements recorded with the AEOS exoscope. We cannot specifically pinpoint the reason for this higher number of unnecessary movements. Based on previous studies regarding this topic, this may be related to the image quality, the depth of field, or the speed of magnification and focus adjustments [1,9,18].

### 4.2. Practical Considerations

In this study, we applied various exoscope systems to perform an experimental microsurgical bypass procedure. This procedure and the experimental setting in itself ask for specific features of the exoscope systems. Firstly, we did not have to take sterility and other surgical participants into consideration in this experimental setting, thereby simplifying the positioning of the exoscopes and the 3D monitor to enable an ideal unobstructed view for the surgeon. In a more complex and sterile surgical setting with additional participants, a scrub nurse and surgical instrumentation tables [11], the bulkiness of the KINEVO exoscopes may complicate its practical use. This is a limitation of this exoscope compared to the other more compact exoscopes, whereas the image quality was evaluated best using the KINEVO.

Secondly, the included procedure is a static procedure, in particular when performed in an experimental setting, demanding only minor adjustments in the vertical plane (i.e., magnification and focus), while no movements are needed in the horizontal plane. This is relevant, as for example the VITOM exoscope was fixed on a fixation bar, thereby impeding movements in the horizontal plane, and thus limiting its application in real life surgical procedures that continuously require major movements of the exoscope. For such procedures, the AEOS and KINEVO may be better as they enable foot pedal-controlled movements in the horizontal plane [18]. Importantly, the results with a more or less fixed magnification and focus setting with the VITOM exoscope, suggest that one benefits from thorough preparations to find the optimal settings for magnification and focus before starting the procedures.

On the one hand, the abovementioned demands of the procedure in this specific experimental setting show that the different exoscopes bring various advantages and limitations to the table. On the other hand, our findings illustrate that the type of procedure, static or dynamic, and the setting complexity (experimental or surgical, number of participants, patient positioning, sterile draping, operating room spatial capacity) also define which type of exoscope system best fits the demands. This is in line with a series by Motov et al. describing the clinical experience of multiple surgeons that started to use an exoscope [25]. In the first few cases, they noticed issues with the location of the exoscope monitor and additional equipment such as fluoroscopy [25].

These and our findings underline the importance of training and preparation before applying a novel tool such as the exoscope in the operating room. Training and preparation not only mean that the surgeon should be acquainted with the settings, features and controls of the exoscopes. Training also includes preparing the optimal practical setup in an experimental setting that mimics the surgical conditions with all involved equipment. Besides this, our findings on the prolonged duration of bypass procedures regardless of the exoscope system emphasize that it takes time and experience to fully get acquainted with novel technical innovations such as the exoscope. In a recent study we have described our experience with the use of the exoscope for resection of a vestibular schwannoma and showed that, with time, practice and experience, surgical time can in fact be reduced compared to the operating microscope [24].

### 4.3. Limitations

This study has several limitations. In this study, only one type of procedure was performed in a highly specific setting. This may influence the outcome of the exoscope systems which does not reflect the quality of an exoscope system perse, but more its suitability for this kind of procedure under these experimental conditions. In particular, this procedure is a static procedure without major movements with the exoscope camera and arm. In real life surgeries such movements are applied continuously and surgical convenience is highly dependent on a fluent and rapid course of these movements. Furthermore, the procedures were performed by one surgeon in order to limit confounding of the results. The inclusion of more than one surgeon would have strengthened the validity of our results. Thirdly, the number of procedures is relatively low which was due to the often-short timespan during which the exoscope was available. In this regard, it is noteworthy that the procedures were performed during the period that the exoscope was available in our center for a clinical test period. Another limitation is that the outcome measures have some subjective judgments; measuring bypass outcomes is more a combination of technical experience and technique than it is reflective of the relative efficacy of the exoscopes. Moreover, reported visualization outcomes are largely subjective and qualitative and do not substantively change the impression that the choice between visualization techniques is also highly subjective and user-dependent. The additional benefits of improved teaching and training residents and medical students by using the exoscope has been described repetitively [26,27], yet was not evaluated in this study.

Regarding the visualization systems, not all commercially available 3D exoscopes were included in this comparison. Due to COVID-19 restrictions we were not able to include the Synaptive Modus V [28]. In addition, Brainpath exoscope system (NICO, Indianapolis, IN, USA) [29], ARveo 8 neurosurgery microscope (Leica Microsystems, Wetzlar, Germany), and MITAKA KV II (Mitaka Co., Tokyo, Japan) [30], and probably some other exoscope systems are available on the market but were not included in our study. We did also not in include a comparison with a conventional microscope as we published about such comparisons previously [1,9] and this would distract from the aim of this study, namely the comparison of different exoscope systems.

## 5. Conclusions

In this study, we showed that all included exoscopes enabled us to perform the same challenging microsurgical procedures with mostly adequate results. Due to the technical and practical differences of the exoscopes, the suitability of an exoscope mostly depends on the demands of the procedure to be performed, and the applicable surgical conditions. Given the specific features of each exoscope, surgeons must consider preprocedural training with the exoscope and thorough preparation of the optimal practical setup of the exoscopic system. With time, practice and experience, the surgeon will get acquainted with the technical demands and practical considerations.

## Figures and Tables

**Table 1:** Practical scale for evaluating the quality of the anastomosis.

	Anastomosis Quality Factor	Value	Points
**T**	Closure time for 20 stitches in 1 mm vessel	<20 min	1
		>20 min	0
**S**	Good distribution of stitches	Yes	1
		No	0
**I**	Thread hidden inside lumen (intima-intima contact)	Yes	1
		No	0
**O**	Width of the orifice (equal or wider than vessel diameter)	Yes	1
		No	0

TSIO-grade is calculated by the sum of points given for each TSIO-quality factor.

**Table 2:** TSIO score per exoscope system.

Exoscope System	TSIO Score					Number of Favorable TSIO Scores *
	0	1	2	3	4	
VITOM	2	1	4	3	-	7/10
KINEVO	-	2	8	-	-	8/10
AEOS	-	1	2	7	-	9/10
ORBEYE	1	1	2	6	-	6/10

* A TSIO score of 3 and 4 is defined as a favorable TSIO score. Abbreviations: TSIO; duration of the procedure (T), distribution of the stitches (S), whether the thread is hidden within the vascular wall (I), and the orifice width (O).

**Table 3:** Unnecessary movements per exoscope system.

Exoscope System	Mean Duration of Bypass Procedure (± SD, Range) in Minutes	Mean Number of Unnecessary Movements (± SD, Range)
VITOM	27.7 (± 1.8, 25–31)	27.0 (± 4.6, 20–35)
KINEVO	29.5 (± 1.8, 26–32)	32.5 (± 9.5, 24–58)
AEOS	26.2 (± 2.7, 21–29)	25.6 (± 5.4, 20–37)
ORBEYE	34.0 (± 3,1, 31–40)	49.2 (± 11.1, 32–69)

Abbreviations: SD; standard deviation.

## Data Availability

Data is available via the corresponding author upon request.
